# Effects of population-related variation in plant primary and secondary metabolites on aboveground and belowground multitrophic interactions

**DOI:** 10.1007/s00049-016-0222-0

**Published:** 2016-10-06

**Authors:** Moniek van Geem, Rieta Gols, Ciska E. Raaijmakers, Jeffrey A. Harvey

**Affiliations:** 1 Department of Terrestrial Ecology, Netherlands Institute of Ecology (NIOO-KNAW), Wageningen, The Netherlands; 2 Laboratory of Entomology, Wageningen University and Research, Wageningen, The Netherlands; 3 Department of Ecological Sciences, Section Animal Ecology, VU University, Amsterdam, The Netherlands

**Keywords:** Aboveground–belowground interactions, Amino acids, *Brassica oleracea*, *Cotesia vestalis*, Glucosinolates, Plant chemistry, *Plutella xylostella*, Primary and secondary metabolites, Sugars

## Abstract

Insects feeding on aboveground and belowground tissues can influence each other through their shared plant and this is often mediated by changes in plant chemistry. We examined the effects of belowground root fly (*Delia radicum*) herbivory on the performance of an aboveground herbivore (*Plutella xylostella*) and its endoparasitoid wasp (*Cotesia vestalis*). Insects were reared on three populations of wild cabbage (*Brassica oleracea*) plants, exhibiting qualitative and quantitative differences in root and shoot defense chemistry, that had or had not been exposed to root herbivory. In addition, we measured primary (amino acids and sugars) and secondary [glucosinolate (GS)] chemistry in plants exposed to the various plant population-treatment combinations to determine to what extent plant chemistry could explain variation in insect performance variables using multivariate statistics. In general, insect performance was more strongly affected by plant population than by herbivory in the opposite compartment, suggesting that population-related differences in plant quality are larger than those induced by herbivory. Sugar profiles were similar in the three populations and concentrations only changed in damaged tissues. In addition to population-related differences, amino acid concentrations primarily changed locally in response to herbivory. Whether GS concentrations changed in response to herbivory (indole GS) or whether there were only population-related differences (aliphatic GS) depended on GS class. Poor correlations between performance and chemical attributes made biological interpretation of these results difficult. Moreover, trade-offs between life history traits suggest that factors other than food nutritional quality contribute to the expression of life history traits.

## Introduction

Plant–insect interactions have long underpinned ecological and evolutionary theory covering vastly different scales, from gene expression to the levels of communities and ecosystems (see Hairston et al. 1960; Root 1973; Futuyma and Agrawal 2009). One of the major shortcomings of much of the research on plant–insect interactions is that until recently most studies focused primarily on the aboveground (AG) compartment. Over the past two decades, however, it has become increasingly recognized that a better understanding of AG processes needs to incorporate biotic interactions occurring in the rhizosphere (Masters and Brown 1992; van der Putten et al. 2001; Bezemer and van Dam 2005). Plant roots, which are essential for the uptake and storage of nutrients, also harbor many mutualists and/or antagonists such as nematodes, insects and pathogens (Waisel et al. 1996; Blossey and Hunt-Joshi 2003). Belowground (BG) organisms can play an important role in determining the composition of ecological communities, and, at greater spatial scales, larger ecosystem processes such as productivity and resilience (Wardle et al. 2004).

The outcome of interactions between organisms feeding on either AG or BG plant tissues can be positive, negative or neutral (Masters et al. 1993; van der Putten et al. 2001; Bardgett and Wardle 2003; Blossey and Hunt-Joshi 2003; van Dam et al. 2003; Wardle et al. 2004). Organisms in the AG and BG compartments may indirectly interact with each other through changes in the quality and quantity of the shared plant that are mostly mediated by their feeding on plant tissues (Bardgett et al. 1998; van der Putten et al. 2001; Soler et al. 2012). These biological interactions are to a large extent mediated by plant traits such as the production of phytochemicals. Plants produce primary metabolites such as amino acids and carbohydrates that are essential for growth, development and reproduction (Schoonhoven et al. 2005). They also produce secondary metabolites that play no apparent role in fundamental physiological processes and have been shown to function as a defense against plant antagonists such as pathogens and herbivores (Fraenkel 1959; Iason et al. 2012).

Primary and secondary metabolites are also important for consumers up the food chain because they influence plant quality for herbivores and even higher trophic levels up to the terminal end of the food chain (Scriber and Slansky 1981; Slansky 1986; Awmack and Leather 2002; Harvey et al. 2003; Ode 2006). Primary metabolites in plants, for instance, provide nutrients that are essential for the development of the insects that feed on them. Nitrogen (N) is often a limiting nutrient for herbivores and thus concentrations of N may profoundly affect insect development (Awmack and Leather 2002; Fagan et al. 2002). Secondary metabolites are often repellent or even toxic to attacking herbivores (Scriber and Slansky 1981; Schoonhoven et al. 2005). However, secondary metabolites can also act as feeding or oviposition stimulants for well adapted specialist herbivores (Schoonhoven et al. 2005). In such cases, primary metabolites may be a more important determinant of plant quality for consumers than secondary metabolites.

Herbivory can change concentrations of both primary and secondary chemistry (Gange and Brown 1989; Bezemer et al. 2003; Harvey et al. 2003; van Dam et al. 2005; Johnson et al. 2009). Induction of metabolites can occur at the attack site only (i.e. local response) or throughout the entire plant (i.e. systemic response), which can affect the performance of herbivores feeding on other plant organs in the same or opposite compartment (Masters et al. 2001; Bezemer and van Dam 2005). However, studies investigating the ecological effects of herbivore-induced changes in plant secondary chemistry, especially in response to foliar herbivory, dominate the empirical literature, though the number of studies examining these effects in response to root herbivory is increasing (Blossey and Hunt-Joshi 2003; van Dam 2009). Moreover, there is often considerable intra-specific genetic variation in the expression of secondary metabolites in plants (Hartmann 1996; Agrawal 2004; van Geem et al. 2013) and this variation can also have consequences for colonizing herbivores and their natural enemies (Moyes and Raybould 2001; Newton et al. 2009). The ecological effects of intra-specific variability of both primary and secondary metabolite concentrations in response to belowground herbivory are not well-studied. Comparing the performance of insects on plants of the same species that differ in chemistry and are differentially affected by herbivory may help to understand which chemicals are important for insect growth and this is investigated in this study.

We examined the effect of herbivory by a BG specialist herbivore, *Delia radicum* L. (Diptera: Anthomyiidae), on primary and secondary chemistry in three different populations of wild cabbage, *Brassica oleracea* L. (Brassicaceae). In turn, we studied the effect of BG herbivory on the performance of an AG specialist herbivore, *Plutella xylostella* L. (Lepidoptera: Plutellidae), and its natural enemy, *Cotesia vestalis* Haliday (Hymenoptera: Braconidae). British populations of wild cabbage in Dorset and Devon are known to differ profoundly in their chemical defense profiles (Mithen et al. 1995; Moyes et al. 2000; Gols et al. 2008; Newton et al. 2009; van Geem et al. 2013). As with other members of the Brassicales, cabbage plants produce secondary metabolites known as glucosinolates (hereafter GS; Halkier and Gershenzon 2006). The defense mechanism of plants producing GS entails the enzyme myrosinase, which is stored in separate cells. When cells are damaged, e.g. as a result of herbivore feeding, the GS and myrosinase come into contact with each other, resulting in the hydrolysis of GS into potentially toxic/deterrent breakdown products (Textor and Gershenzon 2009). Little is known about how these wild cabbage populations differ in levels of primary metabolites.

We hypothesized that (1) the performance of the AG trophic chain will be affected by BG herbivory, and (2) this may differ on the three plant populations due to differences in primary and secondary chemistry.

## Materials and methods

### Plants and insects

In this experiment we used three wild cabbage populations located in the UK, in the Dorset area near Swanage. Seeds were collected at Kimmeridge (KIM; 50°35′N, 2°03′W), Winspit (WIN; 50°34′N, 2°02′W) and Old Harry (OH; 50°38′N, 1°55′W). From each site, pooled seeds were collected from more than 20 plants. The seeds were germinated in germination soil (‘Lentse Stekgrond’, Lent, The Netherlands). When the seedlings were 1-week old, they were transplanted into 2-L pots (one plant per pot) filled with potting soil (‘Lentse Potgrond’ no. 4, Lent, the Netherlands). The plants were left to grow for 8 weeks in a glasshouse at 21 ± 1 °C, 16L:8D, r.h. 70 % in large trays (675 × 170 cm) that were automatically flooded for 20 min each day with water and nutrients (NH4 1.2, K 7.2, Ca 4.0, Mg 1.82, NO_3_ 12.4, SO4 3.32, P 1.0, Fe 35.0, Mn 8.0, Zn 5.0, B 20.0, Cu 0.5, Mo 0.5 in mmol/L).

The cabbage root fly (*D. radicum*) is a specialist root-feeding herbivore on members of the Brassicaceae. The larvae can weaken the root system and stem of a plant to such an extent that the plant may wilt and die. Adult females use GS to locate their host plants for oviposition (Roessingh et al. 1992).

The University of Rennes, France, provided root fly pupae to start a root fly culture at the NIOO in Wageningen. The French colony was started in September 2009 with root flies collected in the field (Le Rheu, Brittany, France, 48°07016″N, 01°47041″W). At the NIOO, cabbage root fly larvae were reared in a climate room (21 ± 1 °C, 50–70 % r.h., L16:D8) on a mixed diet of sugar, nutritional yeast and milk powder (1:1:1). Water was provided through wet filter paper. Adult root flies were offered fresh pieces of turnip/rutabaga on moist filter paper in a Petri dish for females to oviposit on. After one day, the Petri dish was removed and the eggs were collected by rinsing the pieces of turnip/rutabaga with water and collecting the eggs in a fine sieve. The eggs were then spread on top of intact turnips which were placed in plastic containers filled with coarse sand in which the larvae could pupate after eating their way through the turnip in about 3 weeks.

Larvae of the diamondback moth *P. xylostella* are, like *D. radicum,* specialist feeders on plant species in the Brassicales, with GS acting as feeding and oviposition stimulants (Talekar and Shelton 1993). In large numbers the larvae can cause serious damage to plants by defoliation of the shoots. Larvae originated from cabbage fields near Wageningen University and were reared on Brussels sprout plants (*Brassica oleracea* var. gemmifera cv. Cyrus). Larval stages of this insect used in the experiments were obtained by offering cabbage plants for oviposition to adults in the culture maintained at the Laboratory of Entomology, Wageningen University. The eggs were left on the leaves to hatch. Second instar larvae were transferred to predetermined experimental plants (see below).

*Cotesia vestalis* is a solitary koinobiont endoparasitoid wasp, i.e. the female wasp lays a single egg into the body of the host caterpillar which continues feeding and growing during parasitism. When it is fully grown, the mature wasp larva chews its way out of the dying host caterpillar and spins a cocoon. The wasps originated from the same cabbage fields as its host *P. xylostella*. Female wasps used for parasitism originated from a *C. vestalis* culture maintained on *P. xylostella* feeding on Brussel sprout plants at the Laboratory of Entomology.

### Experimental design

Plants of each of the three populations were assigned to one of six treatments: (1) no herbivores, (2) only root flies, (3) only *P. xylostella*, (4) only parasitized *P. xylostella*, (5) root flies and *P. xylostella*, (6) root flies and parasitized *P. xylostella.* There were ten plants per treatment per population and a total of 180 plants. Each individual plant was covered with a sleeve net (100 × 66 cm, BugDorm, Megaview Science, Taiwan) to confine the insects to their respective plants.

Plants from treatments with root flies (treatments 2, 5 and 6) were each inoculated with eight newly hatched L1 root fly larvae. Sixteen and seventeen days after inoculation with root fly larvae, non-parasitized *P. xylostella* and parasitized larvae (15 L2 larvae per plant), respectively, were placed on the assigned plants. Second instar *P. xylostella* larvae were individually parasitized by *C. vestalis* females by offering hosts to wasps in a vial until insertion of the ovipositor was observed. Insects were allowed to move and feed freely on their host plants until they had completed their larval development.

Pupae from *P. xylostella* were collected one day after they had pupated and placed individually in labeled glass vials. As soon as an adult emerged, the date and time of eclosion and its sex were recorded and the vial was placed in a freezer to kill the adult. Adults were dried in an oven at 70 °C for three days to determine their dry body mass. Cocoons of *C. vestalis* were collected and processed similarly as described for the adult moths. One day after most of the moths or wasps had pupated, leaves of the plants were sampled for chemical analysis (see below). Emerged adult root flies were counted to determine their survival. In addition, sex, body mass and development time were recorded. Following root fly eclosion, the roots were sampled for chemical analysis.

### Chemical analyses

Six leaves were randomly sampled per plant from all plants used in the experiments including control plants that had not been exposed to herbivory. All sampled leaves from plants exposed to *P. xylostella* showed signs of caterpillar feeding damage. Using a cork borer, leaf discs (Ø 1 cm) were excised from the leaves. To sample similar amounts of leaf tissue per surface area, 1 disc was sampled from young, small leaves and 2 discs were sampled from older, larger leaves. For each individual plant the discs were pooled, wrapped in tin foil, labeled and immediately frozen in liquid nitrogen. All the samples were freeze-dried and pulverized with a grinding machine (Retsch, type MM 301). The pulverized material was then weighed into 2-mL Eppendorf tubes (50 ± 2 mg).

To collect root material, roots of all plants were removed from the soil. After rinsing with water to remove soil, the roots were placed in paper bags and stored at −20 °C until further processing. The roots were freeze-dried for 4 days and then cut into small pieces of which both thick and thin roots were collected for chemical analysis. These samples were ground to powder with the grinding machine. For quantification of GS, amino acids and soluble sugars, one global 70 % methanol extraction was conducted (see Van Geem et al. 2015).

*Glucosinolate analysis* GS were desulfated with sulfatase (Sigma type H-1 from Helix pomata) on a DEAE-Sephadex A25 column, and separated by high-performance liquid chromatography (HPLC) with an acetonitrile–water gradient (2–65 % acetonitrile from 0 to 30 min; flow 0.75 mL/min; column temperature; 40 °C) on a reversed phase Alltima C18 column (150 × 4.6 mm). The GS were detected with UV diode array at a wavelength of 229 nm. Sinigrin at five different concentrations (63–625 µM) was used as an external standard for the quantification of the GS. Individual GS were identified based on their retention times and UV spectra compared to those of the standards (EC Community Bureau of Reference, Brussels, Belgium, BCR-367R). Final concentrations (nmol/mg) were calculated by correcting for the volume and dry mass of the extract and original tissue. For a more detailed description of the method see van Dam and Raaijmakers (2006).

*Soluble sugar analysis* Sugars were separated using ion-exchange HPLC with a CarboPac PA1 main column (2 × 250 mm) and a CarboPac PA1 guard column (2 × 50 mm). An isocratic gradient mixture of 10 % 1 M NaOH in water at a flow rate of 0.25 mL/min and a column temperature of 20 °C was used to separate the sugars. For a detailed description of the method see van Dam and Oomen (2008). A reference solution (10 ppm) containing sorbitol, mannitol, trehalose, sucrose, melibiose, glucose and fructose was diluted to obtain 2.5, 5 and 7.5 ppm calibration standards for construction of a reference curve. Quantification was based on these reference curves.

*Amino acid analysis* Amino acids were separated using ion-exchange HPLC with an AminoPac PA10 main column (2 × 250 mm) and an AminoPac PA10 guard column (2 × 50 mm) and were separated with a tenary gradient. The method is described in more detail in van Dam and Oomen (2008). The Sigma AA-S-18 amino acid standard (Sigma, St Louis, MO, USA) containing 17 amino acid was supplemented with asparagine, glutamine and tryptophane (2.5 mM/mLeach) to prepare a calibration curve ranging from 1 to 8 mM for each amino acid, except for cysteine, which had a range of 0.5–4 mM.

### Statistical analyses

We compared the effects of AG herbivory on the performance of the BG herbivore and vice versa on the three cabbage populations in separate analyses for each insect. Survival data were analyzed using logistic regression with plant population and presence/absence of the second herbivore as fixed factors. Development time and adult body mass were analyzed using a mixed model approach with population, herbivore treatment and sex (of the focal herbivore) as fixed factors and cage ID as a random factor to account for the fact that the data obtained from insects developing within the same cage were not independent. Estimation of the model terms was based on Restricted Maximum Likelihood (REML). When any of the factors was significant, Tukey–Kramer multiple comparison tests were performed to analyze contrasts between factor levels.

ANOVA was used to reveal differences in total metabolite concentrations among the plant populations and in response to herbivory. Where needed, the data were log-transformed or square-root-transformed to meet assumptions of normality and homogeneity of variance. In addition to total concentrations of all GS combined, total concentrations were also compared for the three GS classes, indole, aromatic and aliphatic GS, respectively (Halkier and Gershenzon 2006). In many leaf samples the aromatic GS nasturtiin could not be detected and, therefore, this GS class was not compared in leaf tissues. Univariate analyses were conducted using SAS, version 9.3 (SAS Institute Inc., Cary, NC, USA).

Using multivariate redundancy analysis (RDA), a constrained principal component analysis, we determined whether tissues of plant populations and responses to herbivory treatments could be separated based on the chemical profiles. Concentrations (log-transformed and mean centered) of individual chemicals served as variables in the analyses. Data were analyzed separately for the three groups of metabolites (amino acids, sugars, GS) and two tissue types (leaves and roots) (six analyses in total). We used multivariate partitioning of variance analysis to determine how much of the variation in the chemistry variables was uniquely explained by the two factors plant population (KIM, WIN, OH) and herbivory treatment (C = control, AG = only AG herbivory present, BG = only BG herbivore present, AG + BG = both AG and BG herbivore present), respectively. The multivariate counterpart of the ordinary *F* ratio in univariate statistics was calculated using the sums of squares totaled across all response variables to yield the *H*_0_ *F* statistic, which is referred to as a pseudo *F* statistic. Monte Carlo permutation (default setting of 499 permutations) tests were used to determine whether model terms (plant population, plant herbivore treatment or their interaction term) were significant or not (Lepš and Šmilauer 2003).

To determine how much of the variation in the insect performance variables (development time and biomass of each sex and survival to adulthood) could be explained by the variation in plant chemistry we also used RDA. To reduce ‘noise’ levels in the data set we only included those chemicals for which the relation relationship with at least one of the performance variables was significant based on Spearman’s rank correlation tests. Outliers in the adult body mass data for *D. radicum,* i.e. those values that fell outside the range of mean ± 3 × standard error, were removed prior to analysis.

## Results

### Insect performance

**Fig. 1:**
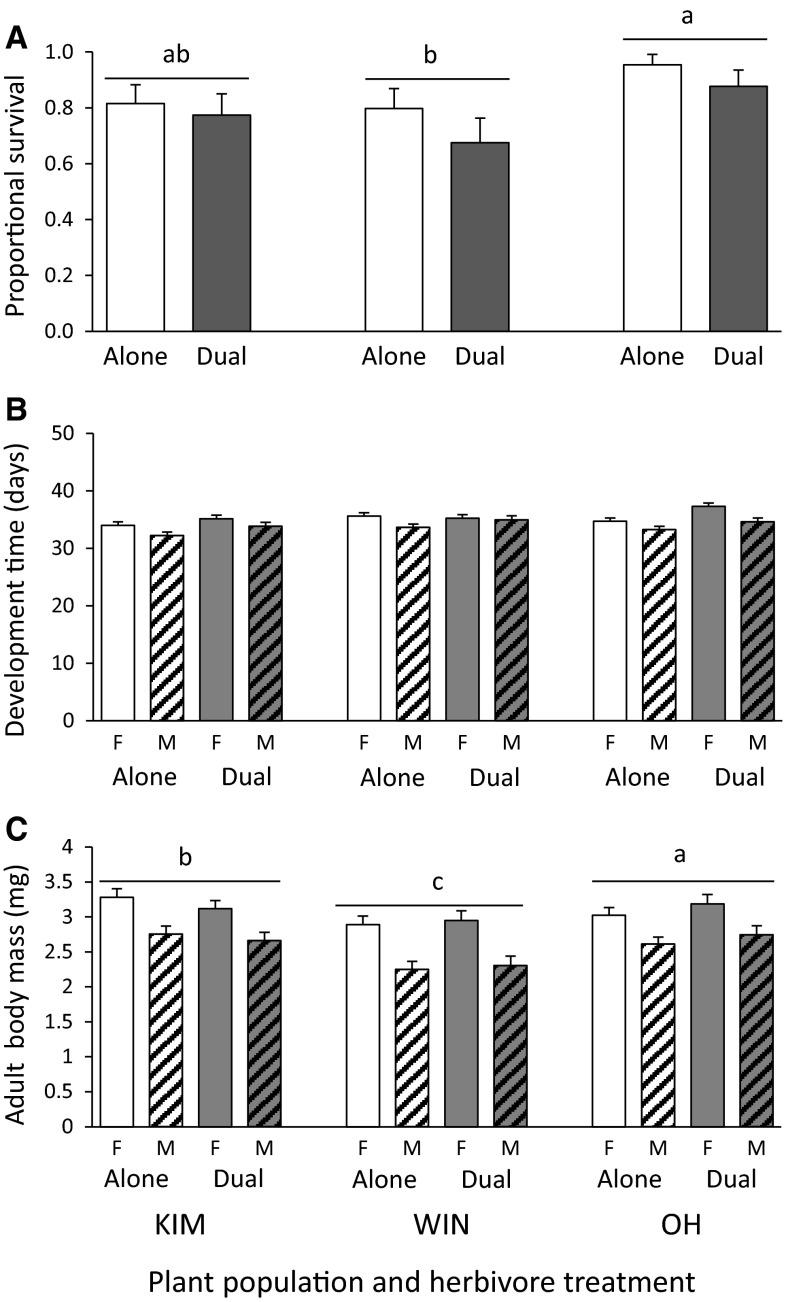
Mean (±SE) proportional survival (**A**), development time (**B**) and adult biomass (**C**) of *Delia radicum* on wild cabbage plants originating from three populations (KIM, WIN and OH) that were reared in the absence (=Alone, *white bars*) or presence of the AG herbivore *P. xylostella* (=Dual, *grey bars*). *Open bars* represent the data for the females and *dashed bars* the data for the males. For the results of the statistical analyses of the main factors and their interaction terms see Table 1. Significant differences (*P* < 0.05) between treatments are indicated by *different letters* based on Tukey–Kramer multiple comparison tests

Survival and body mass of the root fly *D. radicum* was affected by plant population, but not by herbivory treatment, whereas development time was affected by herbivory treatment and not by the population on which the insects had been reared (Table 1). Survival and body mass of *D. radicum* were significantly higher on OH than on WIN plants (Fig. 1A, C). Root flies also attained more biomass on KIM than on WIN plants, whereas survival was similar on these two plant populations. Development time was extended when the plants were also exposed to the AG herbivore *P. xylostella* (Fig. 1B). Females developed slower but attained more biomass than the males (Table 1; Fig. 1B, C).

**Table 1:** Results of the statistical analyses of the insect performance data analyzed per species

Factor	Population (1)		Treatment (2)		Sex(3)		Pop(1)*Treat(2)		Pop(1)*Sex(3)		Treat(2)*Sex(3)		(1)*(2)*(3)	
Insect species	Stat_(df’s)_	*P* value	Stat(_df’s)_	*P* value	Stat(_df’s)_	*P* value	Stat_df’s_	*P* value	Stat_(df’s)_	*P* value	Stat_(df’s)_	*P* value	Stat_(df’s)_	*P* value
Response variable														
*D. radicum*														
Survival^a^	***6.67***_(***2)***_	***0.036***	0.20_(1)_	0.16			0.66_(2)_	0.72						
Development^a^	2.63_(__2,54.7)_	0.08	***7.98***_***(1, 54.7)***_	***0.007***	***60.6***_***(1,390)***_	**<** ***0.001***	0.92 _(__2,54.7)_	0.40	1.85_(2,390)_	0.16	0.52_(1,390)_	0.47	***4.43***_***(2,390)***_	0.012
Biomass^a^	***5.50***_(***2,45.2)***_	***0.007***	0.08_(1, 47.8)_	0.78	***154***_***(1,275)***_	**<** ***0.001***	0.75 _(__2,39.8)_	0.48	2.06_(2,274)_	0.13	0.03_(1,275)_	0.87	0.14_(2,275)_	0.87
* P. xylostella*														
Survival	1.67_(__2)_	0.43	0.31_(1)_	0.58			0.40_(2)_	0.82						
Development	***5.66***_***(2, 53.8)***_	***0.006***	0.08_(1,53.8)_	0.78	0.67_1,540)_	0.41	1.27_(2,53.8)_	0.29	1.08_(2,540)_	0.34	0.58_(1,540)_	0.45	0.70_(2,540)_	0.49
Biomass	***3.28***_***(2, 54.9)***_	***0.045***	1.02_(1,56)_	0.32	***1272***_***(1,575)***_	**<** ***0.001***	1.25_(2,55.9)_	0.29	1.17_(2,574)_	0.31	0.47_(1,574)_	0.49	1.92_(2,574)_	0.15
* C. vestalis*														
Survival	1.58_(__2)_	0.45	0.50_(1)_	0.48			***7.64***_***(2)***_	***0.02***						
Development	***6.30***_***(2,437)***_	***0.002***	***12.1***_***(1,437)***_	**<** ***0.001***	***12.2***_***(1,436)***_	**<** ***0.001***	***6.04***_***(1,437)***_	***0.003***	0.73_(2,436)_	0.48	2.46_(1,436)_	0.12	0.61_(2,436)_	0.54
Biomass	0.32_(2,444)_	0.73	0.38_(1,444)_	0.54	***101***_***(1,444)***_	**<** ***0.001***	0.27_(__1,444)_	0.77	0.38_(2,444)_	0.69	1.00_(1,444)_	0.32	2.98_(2,444)_	0.052

^a^Survival was analyzed using logistic regression analysis, whereas development time and biomass were analyzed using a mixed model approach (REML)

Significant factors are depicted in bold and italicized font

**Fig. 2:**
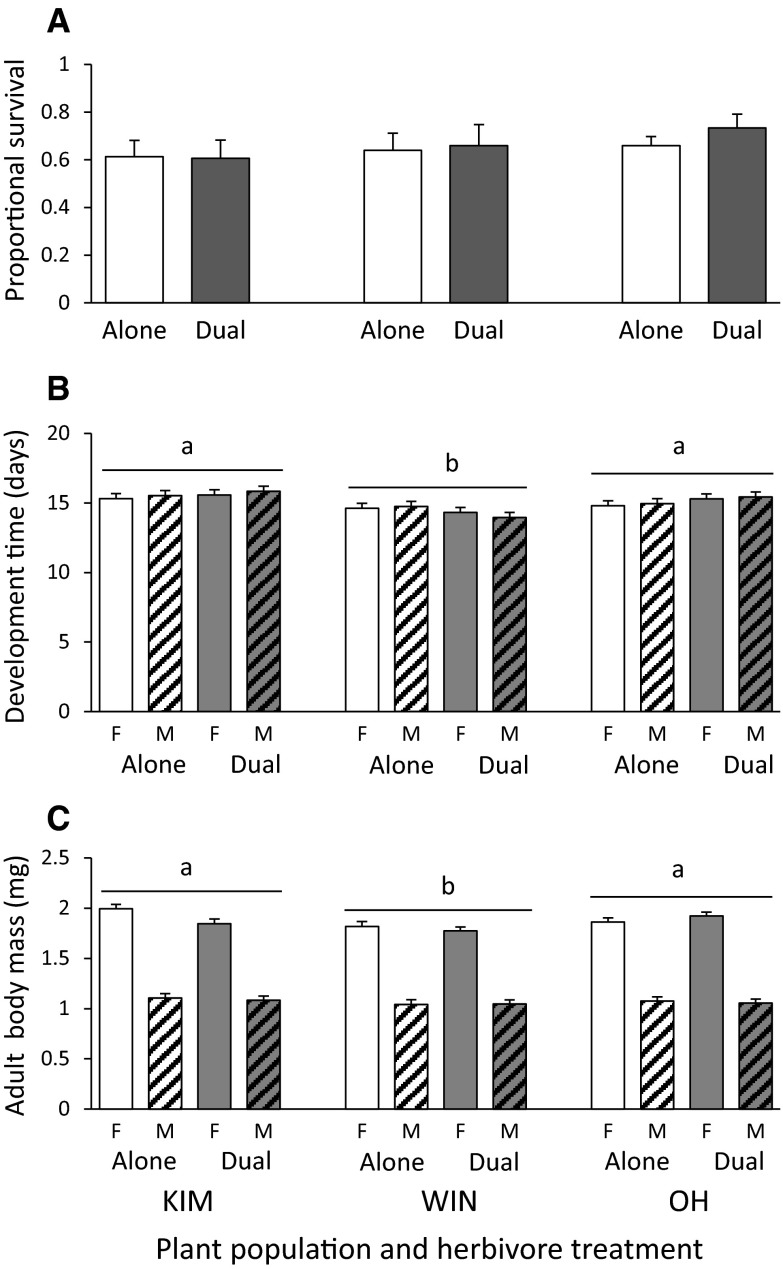
Mean (±SE) survival (**A**), development time (**B**) and adult body mass (**C**) of *Plutella xylostella* on wild cabbage plants origination from three populations (KIM, WIN and OH) that were exposed to the diamondback moth alone (=Alone, *white bars*) or were also exposed to the BG herbivore *D. radicum* (=Dual, *grey bars*). *Open bars* represent the data for the females and *dashed bars* the data for the males. For the results of the statistical analyses of the main factors and their interaction terms see Table 1. Significant differences (*P* < 0.05) between treatments are indicated by *different letters* based on Tukey–Kramer multiple comparison tests

Survival of the AG herbivore *P. xylostella* was similar irrespective of plant population or whether the plants were also exposed to BG herbivory (Table 1). On average 65 % of the caterpillars developed into adults (Fig. 2A). Development time and body mass were significantly affected by plant population and not by BG herbivory (Table 1). Caterpillars developed faster but attained less adult biomass on WIN than on KIM and OH plants (Fig. 2B, C). Adult female moths were significantly heavier (1.75 times on average) than their male conspecifics (Fig. 2C).

**Fig. 3:**
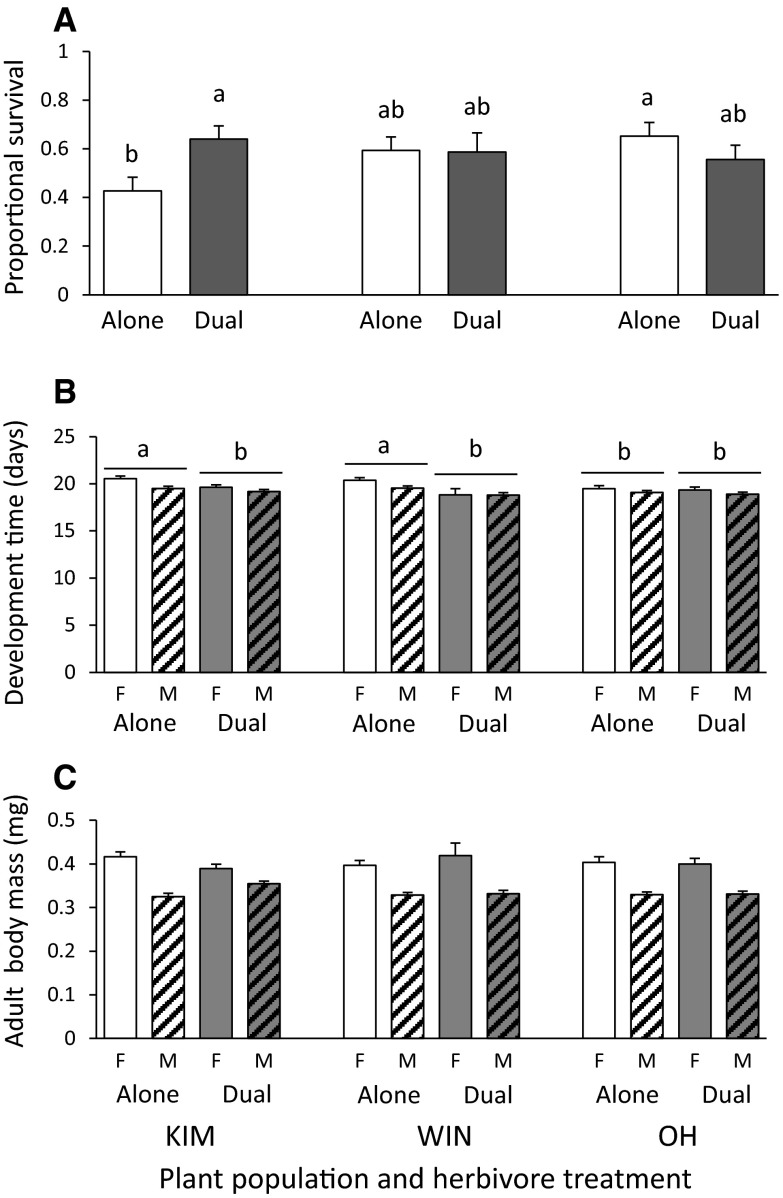
Mean (±SE) survival (**A**), development time (**B**) and adult body mass (**C**) of *Cotesia vestalis* developing in *Plutella xylostella* host larvae on wild cabbage plants originating from three populations (KIM, WIN and OH) that were exposed to the parasitoid-host complex alone (=Alone, *white bars*) or were also exposed to the BG herbivore *D. radicum* (=Dual, *grey bars*). *Open bars* represent the data for the females and *dashed bars* the data for the males. For the results of the statistical analyses of the main factors and their interaction terms see Table 1. Significant differences (*P* < 0.05) between treatments are indicated by *different letters* based on Tukey–Kramer multiple comparison tests

The effect of BG herbivory on survival and development time of the parasitic wasp *C. vestalis* was population specific (Table 1). Survival was lower on KIM plants in the absence of the BG herbivore (Fig. 3A, B), whereas survival was not affected by the presence of the BG herbivore in the other two populations. Development time was shorter on both KIM and WIN plants co-infested with root flies and was similarly low on OH plants, irrespective of root fly presence. Neither plant population, nor root fly presence affected body mass of the emerging wasps (Table 1, Fig. 3C). Female wasps developed slower but grew heavier than male wasps (Table 1; Fig. 3B, C).

### Plant chemistry

**Fig. 4:**
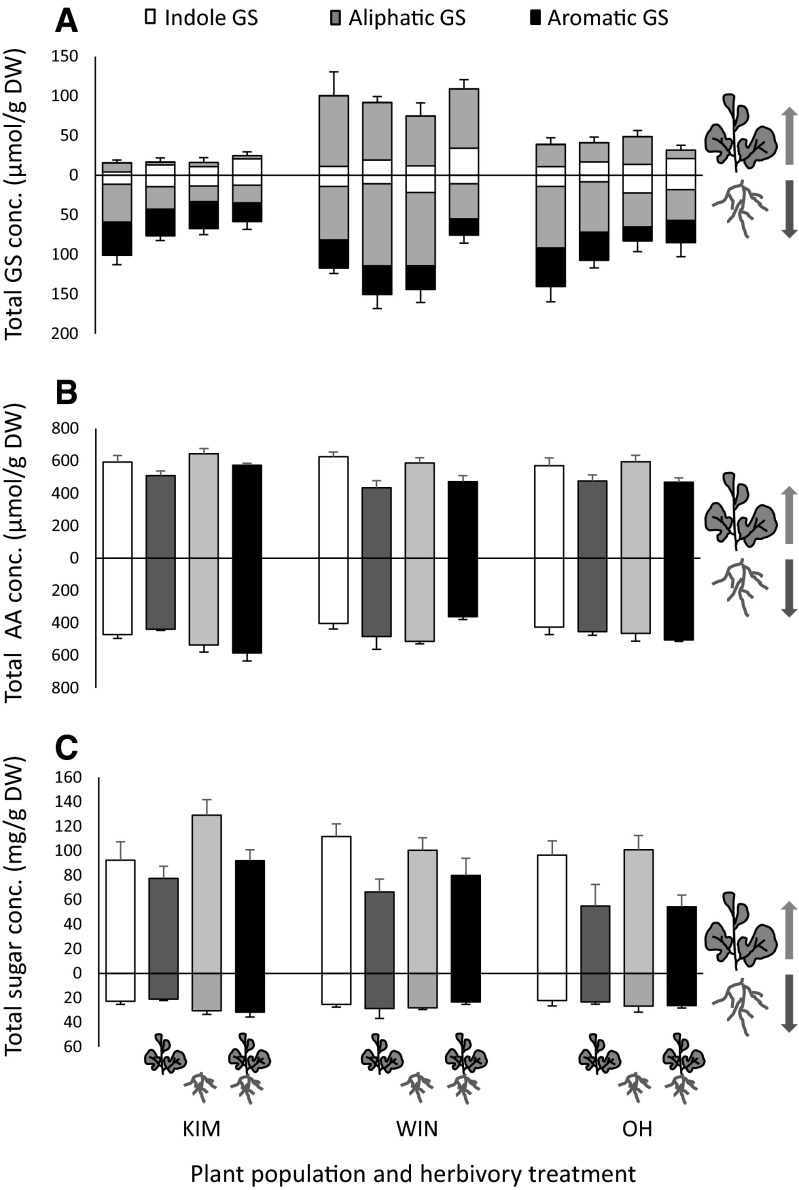
Total concentrations (mean ± SE) of **A** glucosinolates (=GS), **B** amino acids (=AA), and **C** sugars in leaf (data above the *x* axis) and root tissues (data below the *x* axis) of wild cabbage plants originating from three populations (KIM, WIN and OH). On the *x* axis are the four herbivory treatments: control, only aboveground herbivory, only belowground herbivory, and aboveground and belowground herbivory, depicted by the respective plant organs on which the insects feed. The *color shades* in graph (A) refer to the three different GS classes: *white* indole, *grey* aliphatic, *black* aromatic

**Fig. 5:**
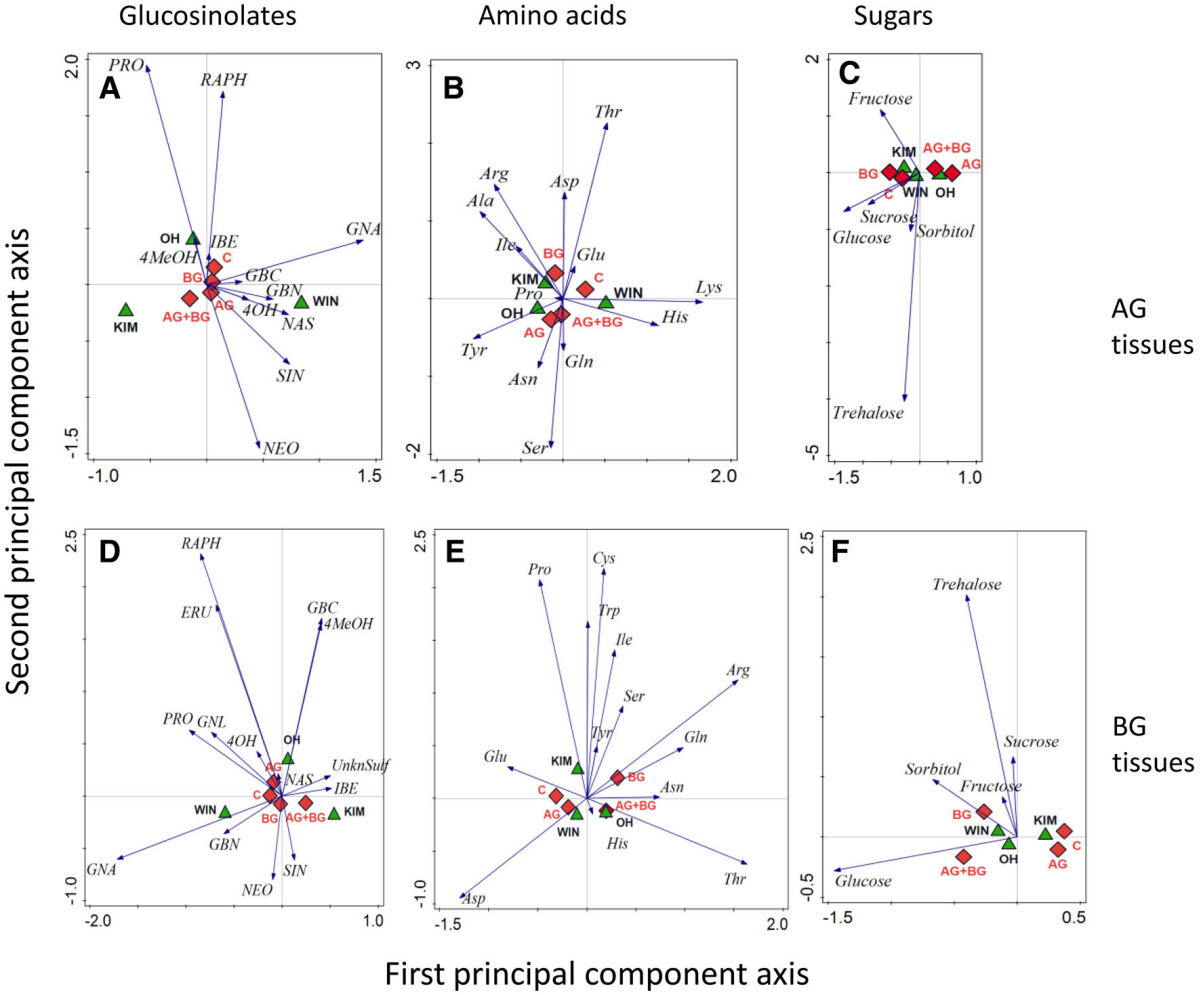
Multivariate RDA analysis of glucosinolates (**A**, **D**), amino acids (**B**, **E**), and sugars (**C**, **F**) in leaf (**A**–**C**) and root (**D**–**F**) tissues of three wild cabbage plants originating from three populations (KIM, WIN and OH) exposed to the AG herbivore alone (AG) the BG herbivore alone (BG), both (AG + BG) or no herbivory (**C**). Graphs summarize the variation in the chemical composition that is uniquely explained by the factors plant population (centrioles are depicted by the *green triangles*) and herbivory treatment (centrioles are depicted by the *red diamonds*). The distance between the symbols approximates the average dissimilarity in chemical composition between the sample classes. Each *arrow* points in the direction of the steepest increase of the values for the corresponding chemical. The angle between *arrows* indicates the degree of correlation between the chemicals and the length of the *arrow* is a measure of fit for the chemical. Projections of the sample classes onto the *arrows* can be used to approximate the average value of that chemical in that class. For the amino acids we used the international IUPAC three letter codes. Abbreviated glucosinolates: aliphatic GS; ERU = glucoerucin, GBN = glucobrassicanapin, GNA = gluconapin, GNL = gluconapoleiferin, IBE = glucoiberin, PRO = progoitrin, RAPH = glucoraphanin, SIN = sinigrin, UnkSul = unknown sulfinyl; indole GS, 4OH = 4-hydroxyglucobrassicin, 4MeOH = 4-methoxyglucobrassicin, GBC = glucobrassicin, NEO = neoglucobrassisin and the aromatic GS NAS = gluconasturtiin. Accompanying statistics are given in Table 1 (Color figure online)

*Glucosinolates* In total fourteen GS were detected, of which three were only present in the roots and not in the leaves (GNL, ERU and an unknown sulfinyl GS, for the full names see Fig. 5). Total GS concentrations differed between the leaves and the roots (ANOVA: *F*_1,178_ = 92.8, *P* < 0.001) with higher concentrations in the roots than in the leaves (Fig. 4a). The GS profiles of leaves and roots were also significantly different from each other (RDA: *F* = 25.5, *P* = 0.001). Concentrations of GBC and GNA were higher in the leaves, whereas concentrations of other GS, especially NAS, the sole aromatic GS, were higher in the roots.

*Glucosinolates in leaves* Total GS and aliphatic GS concentration in the leaves differed significantly among the plant populations, but not in response to herbivory treatments (Table 2). Total GS and total aliphatic concentrations were highest in WIN, intermediate in OH and lowest in KIM plants (Tukey–Kramer, all comparisons *P* < 0.05, Fig. 4A). Contrastingly, total indole concentrations were similar in the three populations, but were affected by herbivory treatment (Table 2). Indole GS were highest in plants dually infested with the AG and BG herbivore (Tukey–Kramer, AG + BG vs. C, *P* < 0.001, AG + BG vs. B, *P* = 0.005, AG + BG vs. AG, *P* = 0.09, Fig. 4A). Indole GS concentrations in plants treated with the AG herbivore alone did not statistically differ from concentrations in plant exposed to the other two herbivore treatments or in control plants.

**Table 2:** Results of the statistical analyses of the chemical data analyzed per tissue type

Factor	Population			Herbivory treatment			Interaction	
Tissue	Stat(_df’s)_	*P* value	%^b^	Stat_(df’s)_	*P* value	%^b^	Stat_(df’s)_	*P* value
Response varibale^a^								
Leaves								
Total GLS	***43.0***_***(2,48)***_	**<** ***0.001***		0.28_(3,48)_	0.84		0.87_(6,48)_	0.53
Total aliphatic GS	***47.2***_***(2,48)***_	**<** ***0.001***		0.84_(3,48)_	0.48		0.69_(6,48)_	0.66
Total indole GS	2.16_(2,48)_	0.13		***7.31***_***(3,48)***_	**<** ***0.001***		0.59_(6,48)_	0.73
GS profiles (RDA)	***31.2***	***0.001***	***49.9***	***2.9***	***0.001***	***7.0***		
Total amino acids	***7.48***_***(2,48)***_	***0.002***		***11.1***_***(3,48)***_	**<** ***0.001***		0.86_(6,48)_	0.53
Amino acid profiles	***6.5***	***0.001***	***16.9***	***3.4***	***0.001***	13.1		
Total sugars	3.10_(2,48)_	0.054		**8.67**_**(3,48)**_	**<0.001**		0.95_(6,48)_	0.47
Sugar profiles	***2.7***	***0.043***	***7.4***	***5.0***	***0.001***	***20.1***		
Roots								
Total GLS	***11.7***_***(2,48)***_	**<** ***0.001***		***8.19***_***(3,48)***_	**<** ***0.001***		***2.77***_***(6,48)***_	***0.02***
Total aliphatic GS	***25.5***_***(2,48)***_	**<** ***0.001***		***8.46***_*(3,48)*_	**<** ***0.001***		***3.55***_***(6,48)***_	***0.005***
Total indole GS	0.65_(2,48)_	0.53		***3.02***_***(3,48)***_	***0.04***		1.23_(6,48)_	0.31
Total aromatic	0.29_(2,48)_	0.75		***5.99***_***(3,48)***_	***0.002***		1.24_(6,48)_	0.30
GS profiles	***11.8***	***0.001***	***28.1***	***2.1***	***0.015***	***7.6***		
Total amino acids	***3.18***_***(2,48)***_	***0.05***		1.89_(3,48)_	0.14		***2.50***_***(6,48)***_	***0.03***
Amino acid profiles	**2.5**	**0.005**	7.6	***2.5***	***0.002***	11.2		
Total sugar	0.32_(2,48)_	0.73		1.22_(3,48)_	0.31		0.88_(6,48)_	0.52
Sugar profiles (RDA)	1.3	0.25	4.0	***3.3***	***0.002***	***14.9***		

^a^Univariate data were analyzed using ANOVA, whereas profiles were analyzed using RDA. Significant factors are depicted in *bold* and *italicized font*

^b^The percentage gives the explained adjusted variation in the RDA analysis

Multivariate analysis of the GS showed that there was a significant effect of plant population and herbivory treatment (Table 2; Fig. 5A). Foliar tissues of OH plants contained relatively high levels of PRO and RAPH, those of WIN plants contained relatively high levels of GNA, whereas in the foliage of KIM plants levels of all GS compounds tended to be lower. In leaf tissues exposed to dual herbivory (AG + BG), concentrations of many aliphatic GS were lowest whereas concentrations of the indole GS GBC, NEO and 4MeOH were highest.

*Glucosinolates in roots.* Total GS and total aliphatic GS concentrations in root tissues differed among the populations and in response to herbivory treatments and also was the interaction between population and herbivory treatment significant (Table 2). Total GS and total aliphatic concentration in KIM and OH plants responded similarly to herbivory treatment (Fig. 4A), though none of the pair-wise treatment comparisons within the KIM and OH populations were statistically significant (Tukey–Kramer; all within-population pair-wise comparisons *P* > 0.05). In WIN plants, total GS and total aliphatic GS concentrations were higher in plants exposed to the single herbivory treatments than in plants exposed to both AG and BG herbivory (Tukey–Kramer AG + BG vs. AG: total GS, *P* = 0.009, total aliphatic GS, *P* = 0.002; AG + BG vs. B, *P* = 0.02 for both total GS and total aliphatic GS). Overall, concentrations of both total and total aliphatic GS ranked KIM < OH ≤ WIN (Tukey–Kramer; total GS: KIM vs. OH, *P* = 0.01; KIM vs. WIN, *P* < 0.001, WIN vs. OH, *P* = 0.20, total aliphatic GS: all pair-wise comparisons *P* < 0.05) and they were lower in dually infested plants than in control plants. Total indole GS and total aromatic GS in the roots were only affected by herbivory treatment (Table 2). Total indole GS were higher in plants exposed the BG alone than in plants exposed to AG herbivore alone (Tukey–Kramer; BG vs. AG, *P* = 0.04, all other pair-wise comparisons *P* > 0.05). Total aromatic GS, i.e. concentrations of NAS, were reduced in root tissue exposed to root herbivory compared to the levels found in control plants (Tukey–Kramer; BG vs. C, *P* = 0.01, BG + AG vs. C, *P* = 0.002).

Multivariate analysis showed a significant effect of plant population and herbivory treatment on the root GS profiles (Table 2; Fig. 5D). As was found for shoot tissues, roots of the WIN population contained relatively high concentrations of GNA. KIM root tissues contained relatively high concentrations of IBE and an unknown sulfinyl GS, and those of OH contained relatively more of the aliphatic GS, RAPH and ERU and the indole GS, GBC and 4MeOH. The differences in relation to herbivory treatment were relatively small. Along the first axis root tissues of plants exposed to both AG and BG and to a lesser extent BG herbivory alone, separated from the other two treatments. Roots of plants exposed to dual herbivory (AG + BG) and root herbivory (BG) contained higher levels of the four indole GS and lower levels of the aromatic GS NAS.

*Amino acids* We detected fifteen amino acids in total, two of which (lysine and alanine) were only found in leaf tissues, whereas cysteine and tryptophan were only found in root tissues in relatively low concentrations. Total amino acid concentrations differed between the leaves and roots (*F*_1,178_ = 31.89, *P* < 0.001), with higher concentrations in the leaves (Fig. 4B). The amino acid profiles of the leaves and roots were also significantly different from each other (*F* = 31.1, *P* = 0.001). Isoleucine and its precursor threonine contributing each c. 40 % to the total foliar amino acid concentrations, whereas root tissues were dominated by serine (c. 75 % of total) and arginine contributed an additional 10 %.

*Amino acids in leaves* Total amino acid concentrations in the leaves differed among the populations and in response to herbivory treatment (Table 2; Fig. 4B). Amino acid concentrations were similar in WIN and OH plants and higher in KIM plants (Tukey–Kramer: WIN vs. OH, *P* = 0.75; KIM vs, OH, *P* = 0.02; KIM vs. WIN, *P* = 0.002. Concentrations of these metabolites were significantly negatively influenced by the presence of the AG herbivore (Tukey–Kramer; C vs. AG, C vs. AG + BG, B vs. AG, B vs. AG + BG, all *P* < 0.05) whereas they were similar in control tissues and tissues exposed to the BG herbivore alone.

The amino acid profiles (multivariate analysis) also differed among the populations and herbivory treatments (Table 2; Fig. 5B). Lysine was only detected in WIN tissues which also contained relatively high concentrations of histidine. Qualitatively, OH and KIM amino acid profiles were more similar to each other than to the profile of WIN plants. Root tissues of OH and KIM plants were characterized by higher relative concentrations of tyrosine, arginine, isoleucine and alanine. In tissues exposed to dual herbivory (AG + BG) and AG alone concentrations of many amino acids, especially threonine, were lower than in control leaves tissues and those of plants exposed to BG alone.

*Amino acids in roots* Population-related differences in the total amino acid concentrations in the roots depended on herbivory treatment (Table 2; Fig. 4B). Among the populations, the response of the plants to dual infestation with both the AG and BG tended to be more variable (AG + BG: KIM vs. WIN, *P* = 0.008 other two comparisons, *P* > 0.05), whereas concentrations were similar in control plants and plant exposed to single infestations with the AG or the BG herbivore, irrespective of plant population (all comparisons, *P* > 0.05). The amino acid profiles (RDA) differed also according to the plant population and the type of herbivory they were exposed to (Table 2; Fig. 5E), but both factors explained relatively little of the total variation (7.6 and 11.2 %, respectively). Tryptophan and cysteine were virtually absent in root tissues of WIN and OH plants, which also contained lower concentrations of proline. Concentration of many amino acids tended to be higher in root tissues exposed to root herbivory (BG, and AG + BG) than in control tissues and plants that had been exposed to AG herbivory alone.

**Fig. 6:**
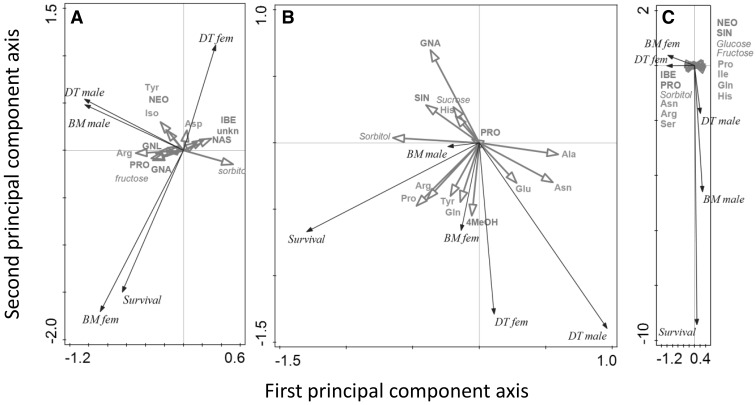
Multivariate RDA analysis of the variance in performance attributes (development time and biomass both for males and females separately (DT male, DT fem, BM male, BM female) and survival) explained by all the chemicals combined (glucosinolates in *red font*, amino acids using the international IUPAC three letter codes in *green font* and sugars in *red italicized font*) for the root fly *Delia radicum* (**A**), the leaf chewer *Plutella xylostella* (**B**) and the parasitoid *Cotesia vestalis* (**C**). For an explanation of the graphs and the abbreviations, see Fig. 5. Accompanying statistics are given in the text (Color figure online)

*Sugars* Five sugars were present in both the leaves and the roots, although the leaf concentrations were considerably higher than the root concentrations (*F*_1,178_ = 296.5, *P* < 0.001, Fig. 4C). The sugar profiles of the leaves and roots were also significantly different from each other (*F* = 169, *P* = 0.001). Glucose and fructose (c. 40 % of total for each sugar) and to a lesser extent sucrose (15 %) were the major foliar sugars, whereas in the roots this was sucrose (c. 80 %) followed by glucose (c. 10 %).

*Sugars in leaves* Total sugar concentrations in the leaves (Fig. 4C) were affected by herbivory treatment and marginally by plant population (Table 2). Total sugar concentrations were lower when the AG herbivore was present, irrespective of BG herbivory (C vs. AG, *P* = 0.007; C vs. AG + AB, *P* = 0.07, BG vs. AG, *P* < 0.001; BG vs. AG + BG, *P* = 0.005). Sugar concentration tended to be highest in KIM leaf tissues. Multivariate analyses showed that the sugar profiles of the leaf tissues were significantly affected by the type of herbivory and also differed among the populations (Table 2; Fig. 5C). The effect of herbivory treatment was stronger than that of plant population. Sugar concentrations were lower in the foliage of plants exposed to AG herbivory alone and in combination with BG herbivory.

*Sugars in roots.* The total sugar concentration in the roots were neither affected by plant population nor by herbivory treatment (Table 2). Multivariate analysis of all individual sugar concentrations combined resulted in a model were some of the variation could be explained by herbivore treatment whereas the effect of population was not significant (Table 2). Many sugar concentrations tended to be higher in root tissues exposed to BG herbivore irrespective of whether there was also AG herbivory (Fig. 5F).

### Insect performance and chemistry

Based on multivariate RDA analysis, variation in performance variables of *P. xylostella* could be significantly explained by variation in primary and secondary foliar chemistry (RDA *F* = 1.7, *P* = 0.036, 26.5 % explained adjusted variation, Fig. 6B), whereas for the root fly and the parasitoid RDA models were statistically not significant (root flies *F* = 1.2, *P* = 0.32, 8.6 % explained adjusted variation, Fig. 6A; parasitoid *F* = 1.3, *P* = 0.33, 12.5 % explained adjusted variation, Fig. 6C). For *P. xylostella*, different performance variables were affected by different chemical attributes. For example, development time of both male and female moths correlated negatively with concentrations of GNA (which means that development time is shorter on plants containing relatively high concentrations of this compound). Survival to adulthood was little affected by concentrations of GNA, but positively by relatively high concentrations of some amino acids such as proline. The variation in *P. xylostella* biomass was more poorly explained by the variation in plant chemistry (indicated by the relatively short length of the biomass arrows in Fig. 6b).

Though the relationships between insect performance and chemical attributes were poor, the difference in relative positions of the performance and chemical attributes suggests that the insect species and even sexes within species responded differently to plant chemistry. For example root fly survival, female biomass and development were positively correlated, but the variation in these variables was poorly explained by variation in root chemistry, whereas performance of the males seems to be explained, to some extent, by concentrations of the analyzed primary and secondary chemistry attributes. Note here the negatively relationship between biomass and development (i.e. heavier wasps took longer to develop). For the parasitoid, the relationship between survival and biomass of the males was positive. Biomass and development of both sexes were negatively correlated, but the relationships between performance and chemistry attributes was different for each sex.

## Discussion

Plant population appeared to have a stronger effect on insect performance than herbivory in the opposite compartment. The effect of AG/BG herbivory was often only significant in specific populations and only affected the root fly and the parasitoid of *P. xylostella* caterpillars but not the AG herbivore itself. Thus, BG herbivory by *D. radicum* exhibited non-linear effects up the food chain, effectively bypassing the AG herbivore, *P. xylostella*, but affecting its endoparasitoid, *C. vestalis* by increasing its survival and reducing its development time. Faster development is considered positive as it reduces the window of vulnerability to natural enemies of the herbivore (Benrey and Denno 1997). Contrastingly, development time of the root fly was negatively influenced, i.e. it was extended, when the AG herbivore was feeding on the same plant. Chemically, differences were found among the populations and in response to herbivory. These were specific for metabolite classes and tissue type. For example, differences in aliphatic GS were most prominent at the population level, whereas differences in indole GS were primarily found in response to herbivory treatment and these were most pronounced when both the AG and BG herbivore where feeding on the plant. Amino acid concentration differed across the populations and decreased locally especially in leaf tissues exposed to the AG herbivore, while sugar concentrations were quite similar among the populations and only differed locally depending on herbivory treatment. Multivariate correlation analyses revealed a poor link between the insect performance and the chemical attributes. Different performance variables often correlated with different chemicals and the patterns in these correlations, if any, were also insect species specific. These results illustrate the complex nature of chemically mediated effects of plant population and herbivory on the performance of higher trophic levels.

For the BG herbivore *D. radicum*, development time was significantly slower in the presence of the AG herbivore *P. xylostella* even though *P. xylostella* were only transferred to the plants in the final larval developmental stage of *D. radicum*. This result reveals that the final stage of larval development of the root fly is highly susceptible to changes in host plant quality that may be mediated by the AG herbivore. The larvae of most holometabolous insect herbivores consume > 80 % of their food in the final instar and are therefore prone to changes in plant quality during this feeding stage. Interestingly, the effects of BG root fly herbivory on the performance of the parasitoid on KIM and WIN plants were positive, which contrasts with results found for *B. nigra* plants dually infested with the same root fly species as in the present study but a different AG herbivore-parasitoid interaction, i.e. *Pieris brassicae* caterpillars parasitized by *Cotesia glomerata* (Soler et al. 2005). In the Soler et al. (2005) study, the herbivore, the parasitoid and even the hyperparasitoid *Lysibia nana* were all negatively influenced by *D. radicum* infestation of the roots. These results suggest that even in closely related plants species AG/BG mediated effects on insects feeding on tissues in the opposite compartment may be highly plant species specific. Moreover, even specialist herbivores, both *P. xylostella* and *P. brassicae* are primarily feeding on plants in the Brassicaceae, may be differentially affected by subtle differences in plant quality.

Overall cabbage population-related effects on insect performance were stronger than changes in plant quality induced by different herbivory treatments. These effects were different for the three insect species. For example, performance of *D. radicum* was better on OH and KIM than on WIN plants. For *P. xylostella* the population-related effects were less clear; though the caterpillars developed faster on WIN than on OH and KIM plants, they also attained lower biomass on WIN plants. In *C. vestalis*, the population effects in KIM and WIN plants depended on the presence of root flies. Cabbage population-related differences in performance of insects have been shown in several other studies with other species of specialist (Harvey et al. 2007, 2011) and generalist (Gols et al. 2008) AG herbivores and their parasitoids. Harvey and Gols (2011), for example, found that development of the generalist herbivore, the cabbage moth *Mamestra brassicae*, was more negatively affected when developing on WIN plants than was development of its parasitoid, *Microplitis mediator*, a pattern which is opposite to what we found here with *P. xylostella* and *C. vestalis* (the parasitoid was positively affected by BG herbivory and the host itself was un affected). *C. vestalis* may be stronger affected by plant derived compounds that are present in the host hemolymph or are stored in body tissues than the hosts are themselves, as *P. xylostella* is well adapted to feed on brassicaceous plant species. Alternatively the immune system of the host is differentially affected by differences in food plant quality with concomitant consequences for the performance of the parasitoid larvae developing in these hosts (Bukovinszky et al. 2009). For the specialist insects studied here, genetic variation in the quality of the plant populations is probably more important than the more subtle within- or between-population effects that are mediated by herbivory in the opposite compartment.

Primary and secondary chemistry in *B. oleracea* differed between leaves and roots, among the wild cabbage populations and, to a lesser extent, among the different herbivory treatments. Total GS concentrations were higher in root than in the leaf tissues, whereas this pattern was reversed for the amino acids and sugars. Although previous work on the same wild cabbage populations found that concentrations of GS were higher in the leaves than in the roots (van Geem et al. 2013), other studies have found that in general GS occur in higher concentrations in the roots than in the leaves (Kaplan et al. 2008; van Dam et al. 2009).The chemical profiles of the leaves and roots were also different in terms of the presence and relative concentrations of individual compounds.

In response to herbivory, changes in root and foliar GS chemistry tended to be most pronounced when both the AG and BG herbivore were feeding on the plants. In response to herbivory by only one herbivore plants responded only locally, that is feeding by the AG herbivore alone affected secondary chemistry only in the leaves and not in the roots and vice versa. In both tissues, concentrations of indole GS increased and concentrations of several aliphatic GS decreased in response to herbivory. The aromatic GS NAS, which was primarily found in root tissues, also decreased in response to root herbivory. Several studies have examined the reciprocal effects of AG and BG herbivory on secondary chemistry in plants. For example, Kaplan and colleagues (2008) found that in tobacco plants AG herbivory only affected leaf chemistry, whereas BG herbivory affected both leaf and root chemistry. Their findings were supported by a meta-analysis on induced defenses in a wide range of plant herbivore systems which showed that generally leaf herbivory induces secondary chemistry only in leaf tissues, whereas root herbivory induces both leaf and root secondary chemistry (Kaplan et al. 2008). Our study showed that within a group of secondary metabolites, the response to herbivory may differ as well. Increases in indole GS in response to herbivory are well documented (Agerbirk et al. 2009), whereas herbivore-induced changes in aliphatic and aromatic GS have been reported to be more idiosyncratic (Hopkins et al. 2009). The defensive function of aliphatic GS is often associated with myrosinase-catalyzed degradation of these compounds following tissue damage (Halkier and Gershenzon 2006).

In addition to population-related differences, changes in amino acids concentrations were only expressed in the tissues that were exposed to herbivory and not in the tissues in the opposite compartment and these effects were more pronounced in leaf than in root tissues. In response to AG herbivory, many amino acid concentration in the leaves decreased, whereas in the roots the response to root fly feeding was more variable and depended on cabbage population. Similarly, Johnson et al. (2009) reported that aphid feeding reduced amino acid concentrations in the leaves, whereas BG herbivory by wireworms had little effect on foliar amino acid concentrations. However, in tomatoes, changes in primary metabolites in response to herbivory by leaf-chewing caterpillars were systemic, i.e. they were also detected in the roots, and they were found to be tissue- and herbivore-specific (Steinbrenner et al. 2011). In a field study with blackcurrant bushes, BG herbivory by weevils increased foliar amino acid concentrations (Johnson et al. 2013). Our results, together with those of other studies, show that whether changes in amino acid concentration are only observed in the tissue that is exposed to herbivory or whether they are expressed systemically depend on the plant species and the identity of the attacking herbivore. In addition, the response may be further influenced by genetic variation within plant species.

Total sugar concentrations and sugar profiles in the leaves and roots differed only marginally among the wild cabbage populations and the herbivory treatments. Sugar concentrations were only locally affected in the tissues that were damaged. AG herbivory decreased total sugar concentrations in the leaves, whereas BG herbivory increased total sugar concentration in the roots. The reduced sugar concentration in the leaves could be the result of leaf damage and an associated reduction in photosynthesis, since sucrose is one of the end products of photosynthesis (Huber 1989). Re-allocation of resources from the damaged plant parts to undamaged plant parts as a reaction to leaf herbivory is a known phenomenon and part of a plant defense mechanism known as tolerance (Rosenthal and Kotanen 1994; Stowe et al. 2000; Orians et al. 2011). However, there was no effect of AG herbivory on root sugar concentrations. The higher root sugar concentrations following BG herbivory could have been the result of regrowth of roots and thus a higher demand for resources.

British wild cabbage plants are interesting in that they only grow in chalky soils in generally rugged coastal habitats; some of these locations are very exposed to prevailing winds whereas others are not. In turn, the exposure of the plants to prevailing winds will certainly affect the ability of herbivores to find and exploit these plants. Of the Dorset populations studied, WIN plants grow in the most secluded location and large populations of herbivores (e.g. larvae of pierid butterflies, cabbage aphids, whiteflies) have been occasionally found on them. On the other hand KIM plants grow along a very exposed eroding cliff face and this location is almost continually buffeted by strong winds along the English Channel; consequently very few insects have been found on these plants (J. Harvey and R. Gols, unpublished observations). Differences in selection pressure by insect herbivores and abiotic factors, both AG and BG may explain some population-related variation in the concentrations of GS and amino acids, which often are precursors in the biosynthesis of many metabolites including GS. Interestingly, population-related difference in herbivore-induced responses were primarily found in root tissues. Glucose, fructose and sucrose as the major products of photosynthesis may be less prone to genetic differentiation.

Multivariate correlations analysis between insect performance and chemistry variables resulted in a significant model for *P. xylostella* only. Different performance attributes correlated with different chemistry attributes. For instance insect development was faster on plant containing high levels of GNA, whereas survival was primarily correlated with levels of specific amino acids. The biological interpretation of these results is difficult, but it suggests that different life history traits are differentially affected by chemical quality traits of the food plant. Experiments with artificial diets in which concentrations of single or a few compounds are varied may determine the relative effect of some nutritional or adverse diet ingredients on insect growth. However, this approach may not help to explain complementary, additive, or antagonistic effects considering the full diet. Metabolomics approaches and multivariate statistics may only have a marginal value in explaining insect performance on plants exposed to different treatments as they do not consider complex interactions between metabolites (Behmer 2009), but they may be used for explorative purposes. Furthermore, in this study we only considered a subset of metabolites which were only measured at a single time point. Most likely, other phytochemicals and variation in morphological traits that were not measured here also affected insect performance. In addition, trade-offs between life history traits such as the investment in reduced development time at the cost of being smaller (e.g. in *C. vestalis*), suggest that other factors than food nutritional quality, determine the expressed value of a life history trait.

In summary, our results highlight the fact that changes in plant quality in response to AG or BG herbivory or both can be relatively subtle and that the effects on the developmental performance of the specialized insects involved are species-specific with visible effects that may ‘jump’ from the plant to the third trophic level. Moreover, for the wild cabbage populations studied here, differences in plant quality between the populations appear to be greater than the herbivore-induced changes in plant quality. This does not mean that within-population variation is not present, but that the variation masks effects mediated by AG and BG herbivores. Furthermore, given that they are all specialists, the insects are probably labile in terms of their ability to deal with differences in plant quality. As the performance of the insects was primarily affected by qualitative differences among the populations, differences in primary metabolites that were primarily induced by herbivory and expressed locally may have contributed little in explaining differences in insect performance. It may be that this variation has more apparent effects on less well adapted organisms, including generalist herbivores, as previous studies have shown (e.g. Gols et al. 2008). Future work aims to explore AG and BG interactions in wild cabbages across a broader range of species as well as to better understand communities associated with the plants in the field.
